# Supplementary material for: Exploring the Diversity of the Thioredoxin Systems in Cyanobacteria
Source: Antioxidants (Basel). 2022 Mar 28;11(4):654. doi: 10.3390/antiox11040654 (PMC9025218; doi:10.3390/antiox11040654)
Supplement: Supplementary file 1 [file antioxidants-11-00654-s001.zip › antioxidants-1632615-supplementary/Suplementario Data v2.pdf]

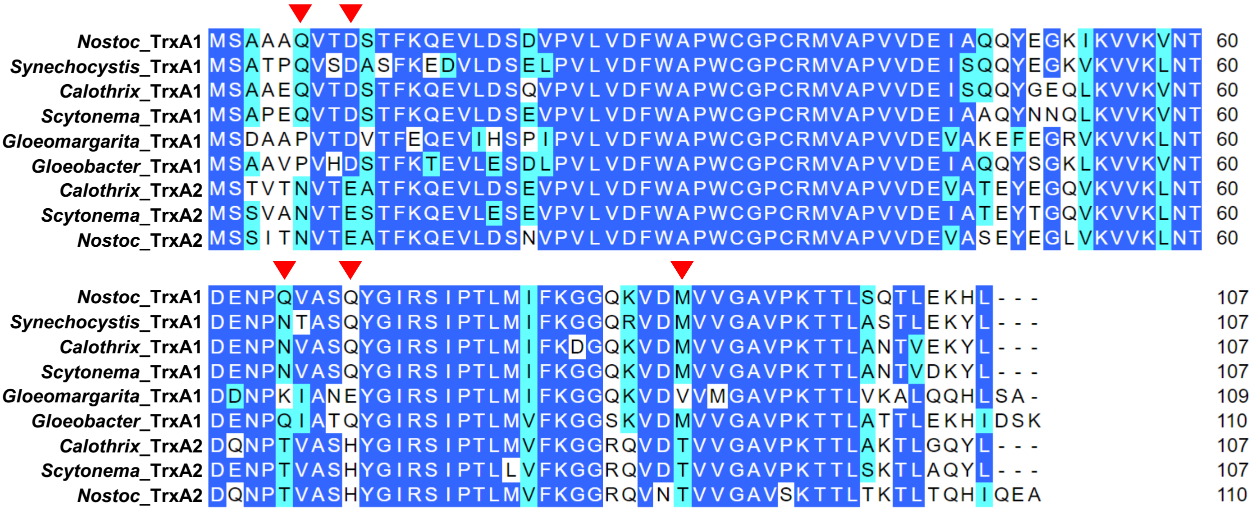

Figure S1. Multiple protein sequence alignment of several TrxA1 and TrxA2. The sequences are indicates with abbreviations of the organism names: *Nostoc* sp. PCC 7120 TrxA1 (Nostoc_TrxA1), *Synechocystis sp.* PCC 6803 TrxA1 (*Synechocystis*_TrxA1), *Calothrix* sp. 336/3 TrxA1 (*Calothrix*_TrxA1), *Scytonema* sp. HK-05 TrxA1 (*Scytonema*_TrxA1), *Gloeomargarita lithophora* TrxA1 (*Gloeomargarita* _TrxA1), *Gloeobacter violeaceus* TrxA1 (*Gloeobacter*_TrxA1), *Calothrix* sp. 336/3 TrxA2 (*Calothrix*_TrxA2), *Scytonema* sp. HK-05 TrxA2 (*Scytonema*_TrxA2), and *Nostoc* sp. PCC 7120 TrxA2 (Nostoc_TrxA2). The red triangles indicate the unique amino acids that are specific to TrxA2. Blue and cyan color indicate identity or similarity, respectively.

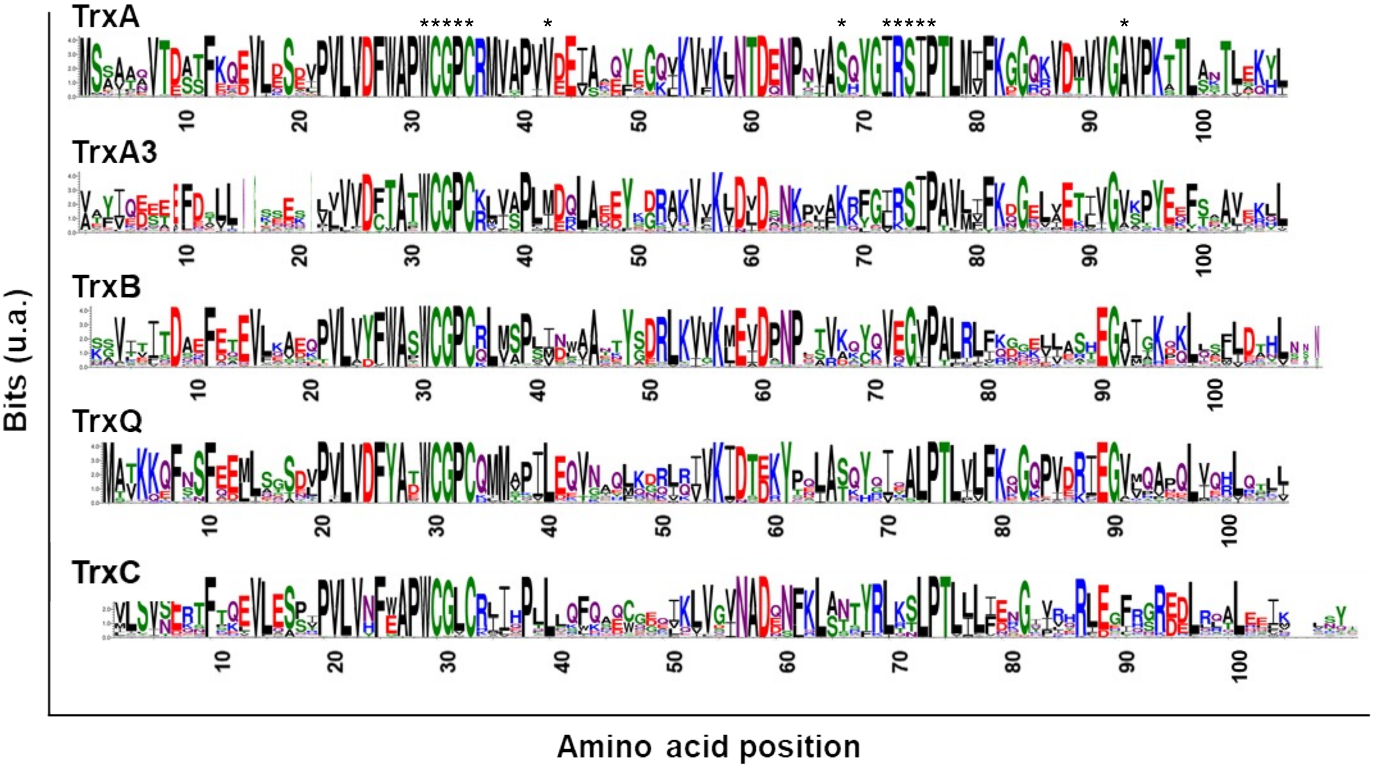

Figure S2. Sequence conservation of TrxA1/2, TrxA3, TrxB, TrxQ and TrxC. Logos were built using blast at IMG and NCBI databases, and manually curated in all cases. The sequences were aligned with Muscle algorithm and the aligmnment were generated with Weblogo3. The height of each amino acid code for each position indicate the frequency at that position. The numbering corresponds to the respective position in the Trxs of *Nostoc* sp. PCC 7120. Essential amino acids for the formation of the FTR-TrxA complex are marked with asterisks according to Dai et al., 2007.

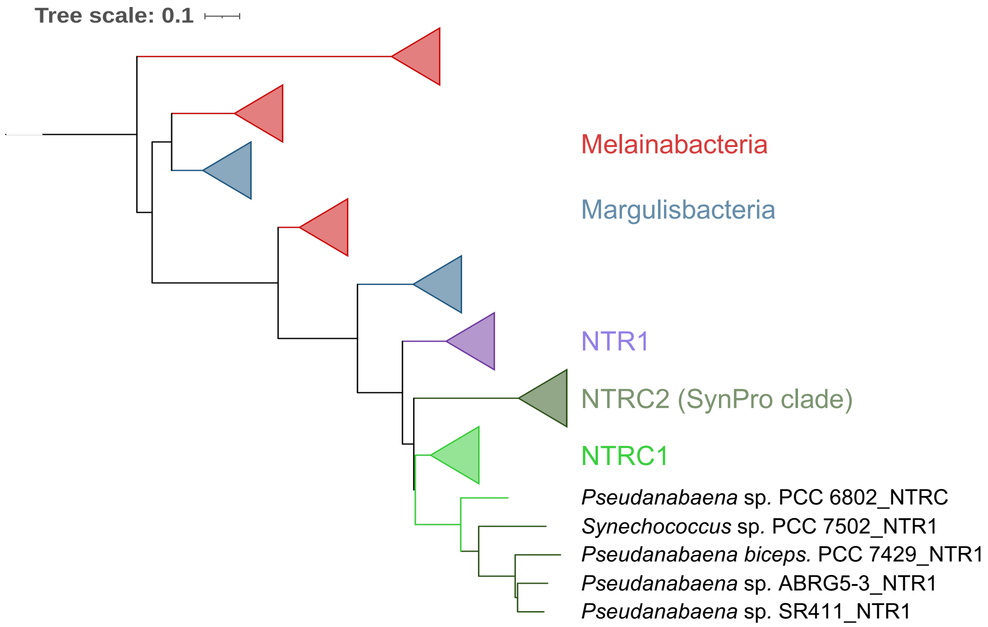

Figure S3. Phylogenetic tree related to NTRC and NTR1 present in Cyanobacteria and close relatives.

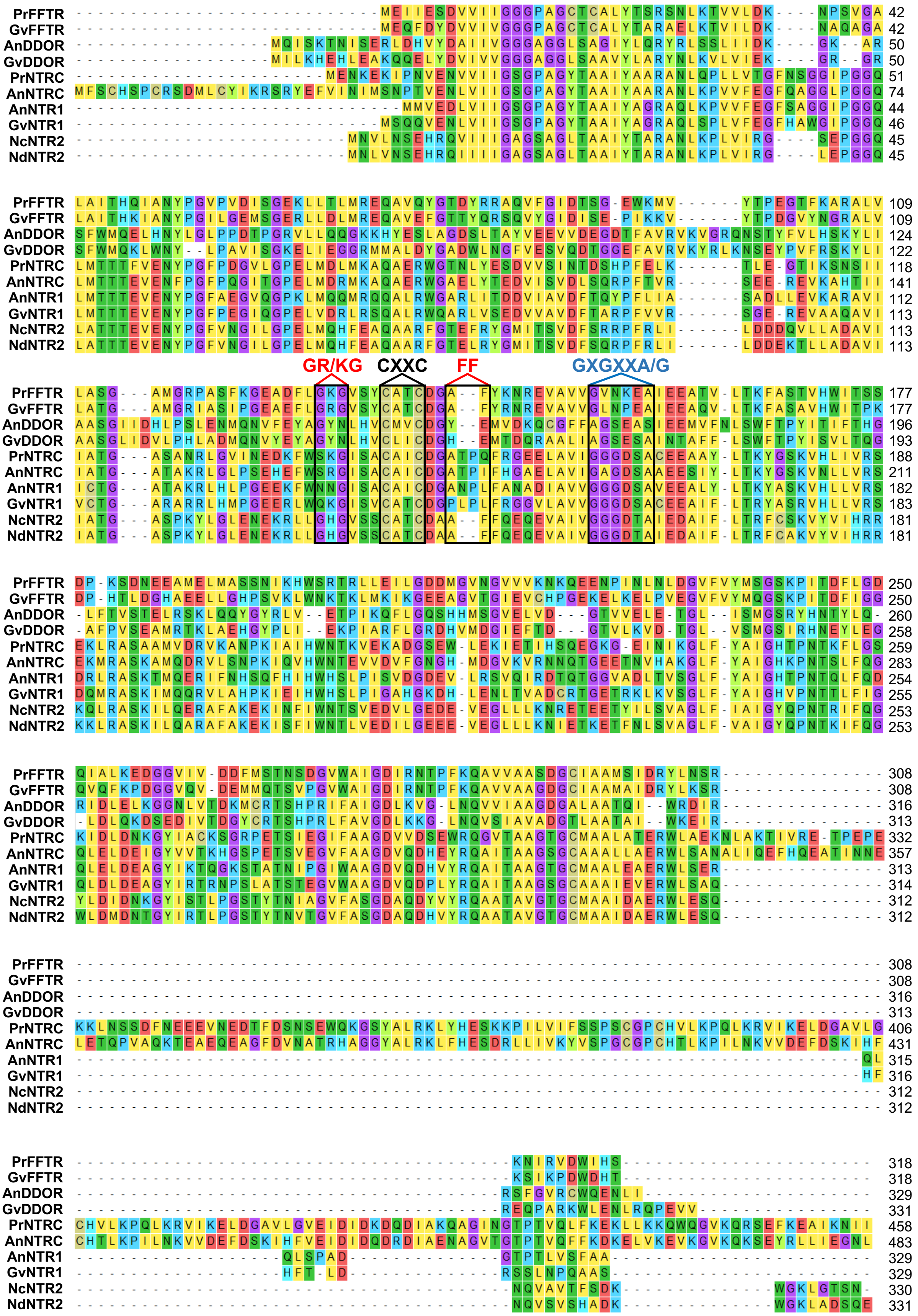

Figure S4. Multiple alignment of full protein sequence of several TR-related enzymes. The sequences are indicated with abbreviations of the organism names as in Figure 3B.

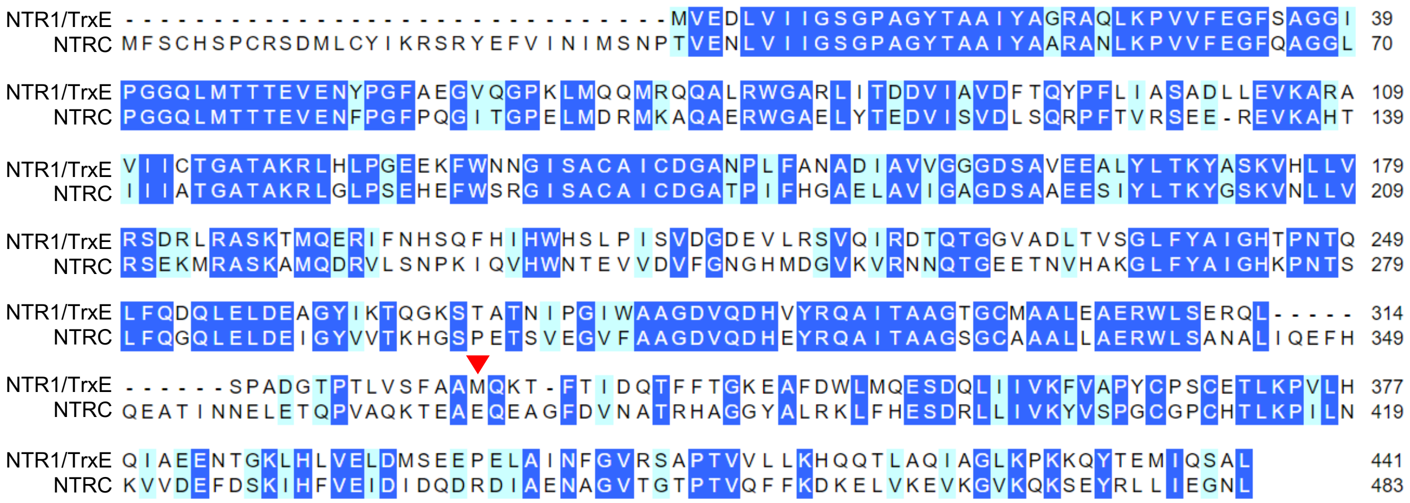

Figure S5. Alignment of the chimeric sequences NTR+TrxE and NTRC. The sequences used correspond to NTRC, NTR1 and TrxE of *Nostoc* sp. PCC 7120. The red triangle indicates the start of the TrxE fragment in the hypothetical chimeric protein. Blue and cyan color indicate identity and similarity, respectively.

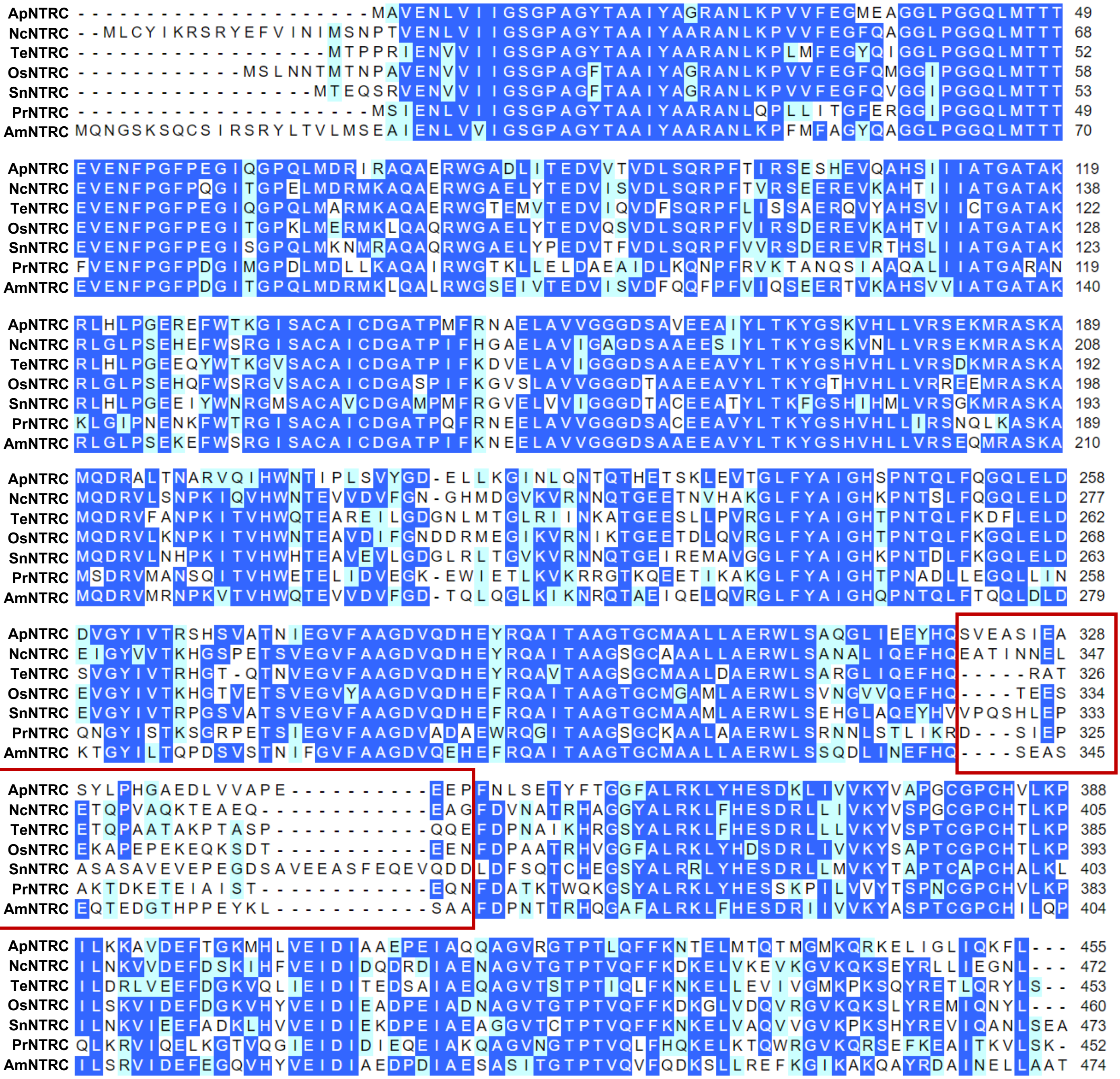

Figure S6. Multiple protein sequence alignment of several NTRC enzymes. The sequences are indicates with abbreviations of the organism names: *Anthocerotibacter panamensis* NTRC (ApNTRC), *Nostoc* sp. PCC 7120 NTRC (NcNTRC), *Thermosynechococcus elongatus* BP-1 NTRC (TeNTRC), *Oscillatoria acuminata* PCC 6304 NTRC (OsNTRC), *Synechococcus* sp. JA-3-3Ab NTRC (SnNTRC), *Prochlorococcus marinus* SS120 NTRC (PrNTRC) and *Acaryochloris marina* MBIC11017 NTRC (AmNTRC). Blue and cyan color indicate identity and similarity, respectively. Red box indicates the region between the NTR and Trx domains.

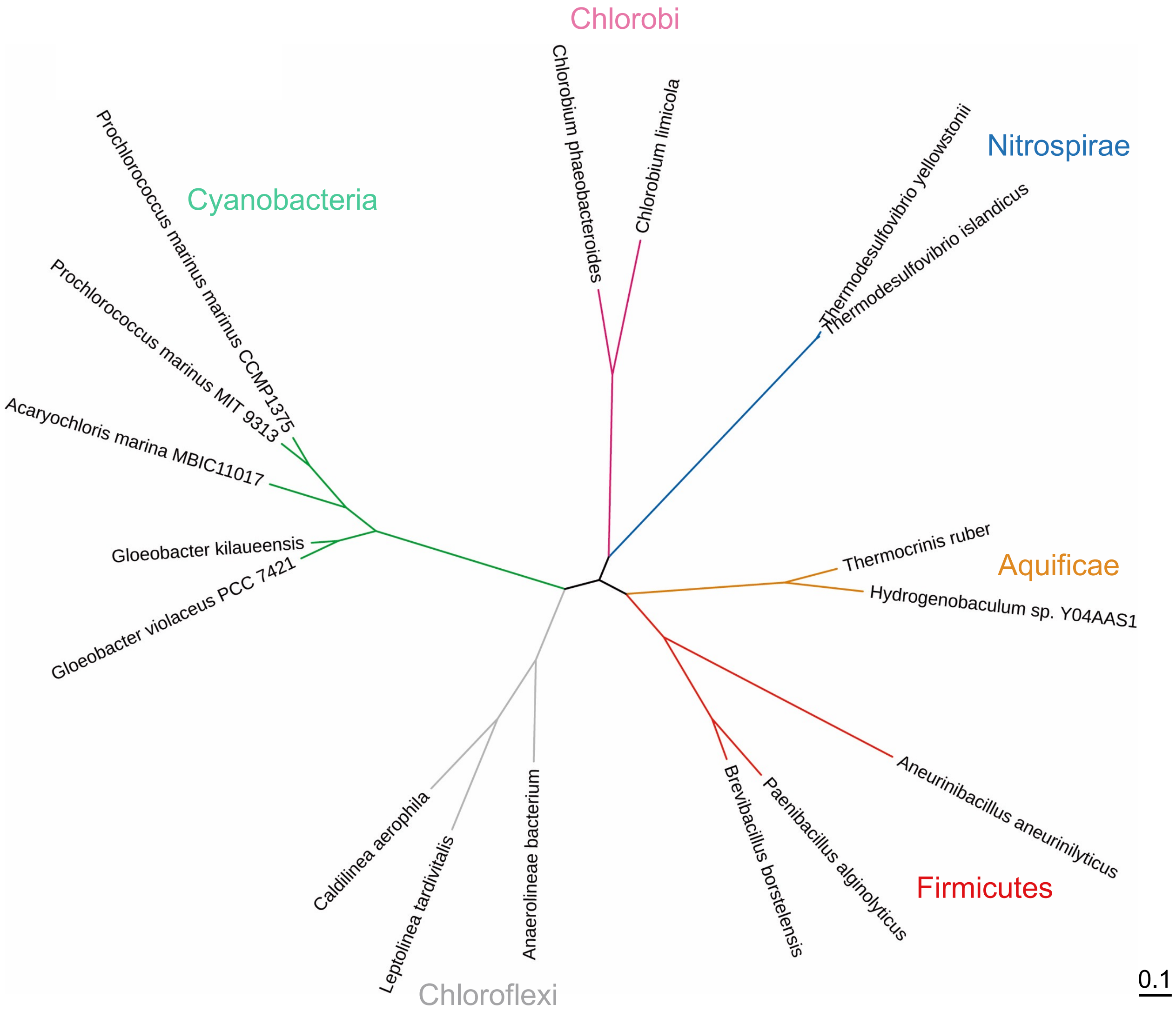

Figure S7. Unrooted tree of FFTR amino acid sequences identified in the IMG and NCBI databases. Branches are colored to represent FFTR amino acid sequences found in the different bacterial phyla: Cyanobacteria (green), Chlorobi (purple), Nitrospirae (blue), Aquificae (brown), Firmicutes (red), and Chloroflexi (grey). Scale bar represents number of substitutions per site.
